# 

**Published:** 1837-10

**Authors:** 


### CONTENTS OF No. VIII.

OF THE

### British and Foreign Medical Review.

OCTOBER, 1837.

---

### PART FIRST.—Analytical and Critical Reviews.

PAGE

ART. I.—1. A Treatise on the Diagnosis and Treatment of Diseases of the Chest. Part I. Diseases of the Lung and Windpipe By WM. STOKES, M.D. &c. . 285

2. Notes et Additions au Traité de l'Auscultation Médiate de LAENNEC. Par M. MERIADEC LAENNEC, D.M.P. &c., et M. ANDRAL, Professeur à la Faculté de Médecine de Paris, Médecin de l'Hôpital de la Charité, &c. . . ib.

Notes and Additions to the Treatise on Mediate Auscultation of LAENNEC. By M. MERIADEC LAENNEC, M.D. Paris, &c.; and M. ANDRAL, Professor of the Faculty of Medicine of Paris; Physician to the Hospital of la Charité, &c.

3. Observations on the Surgical Pathology of the Larynx and Trachea, &c. By WM. HENRY PORTER, A.M. &c. . . . . ib.

4. A Treatise on the Diseases and Injuries of the Larynx and Trachea, &c. By FREDERICK RYLAND, Surgeon to the Town Infirmary, Birmingham . ib.

ART. II.—La Système Lymphatique, considéré sous les Rapports Anatomique, Physiologique, et Pathologique. Par G. BRESCHET . . . 325

The Lymphatic System, considered in Relation to its Anatomy, Physiology, and Pathology. By G. BRESCHET.

ART. III.—Guy's Hospital Reports. No. IV. April, 1837. Edited by G. H. BARLOW, M.A. and L.M., and J. P. BABINGTON, M.A. &c. . . . 349

Mr. KEY's Practical View of Lithotrity; with Remarks on the Lateral Operation of Lithotomy . . . . . . 350

Dr. ADDISON's Observations on the Diagnosis of Pneumonia . . 356

Mr. TAYLOR's Two Cases of Fatal Poisoning by Arsenious Acid, &c. . 358

Mr. KING on the Safety-Valve Function of the right Ventricle of the Human Heart, 364

Mr. BRANSBY COOPER's Experimental Inquiry respecting the Process of Reparation after simple Fractures of Bones . . . . 367

Dr. ASHWELL's Reports of Obstetric Cases, with Observations . . 369

Dr. HODGKIN's Description of a remarkable Specimen of Urinary Calculus . 375

Dr. BRIGHT on the Diagnosis of Tumours situated at the Basis of the Brain . 376

ART. IV.—Ueber den Zustand der Heilkunde und ueber die Volskrankheiten in der Europäischen und Asiatischen Türkei. Ein Beitrag, &c. Von FRIEDR. WILHELM OPPENHEIM, Doctor der Medicin und Chirurgie, &c. . 380

An Essay on the State of Medicine, and on the prevailing Diseases of European and Asiatic Turkey. By F. W. OPPENHEIM, M.D.

ART. V.—1. Practical Observations on various Subjects relating to Midwifery. By JAMES HAMILTON, M.D. F.R.S.E. &c. . . . . 398

2. Observations on the Artificial Dilatation of the Mouth of the Womb during Labour, and upon Instrumental Delivery, &c. By ROBERT COLLINS, M.D. ib.

3. An Inquiry into the Management of the first Stage of Labour. By E. W. MURPHY, M.D. . . . . . . ib.

4. Facts and Cases in Obstetric Medicine; with Observations on some of the most important Diseases incident to Females. By J. T. INGLEBY, Esq. . ib.

ART. VI.—Untersuchungen zur Physiologie und Pathologie. Von Dr. FRIEDRICH NASSE und Dr. HERRMANN NASSE . . . . 414

Physiological and Pathological Researches. By Dr. F. and Dr. H. NASSE.

ART. VII.—Isis Revelata: an Inquiry into the Origin, Progress, and Present State of Animal Magnetism. By J. C. COLQUHOUN, Esq. Advocate, F.R.S.E. 441

PAGE

ART. VIII.—An Exposition of the Signs and Symptoms of Pregnancy, the Period of Human Gestation, and the Signs of Delivery. By W. F. MONTGOMERY, M.D. 448

ART. IX.—A Treatise on the Malformation, Injuries, and Diseases of the Rectum and Anus. By GEORGE BUSHE, M.D. . . . . 467

ART. X.—An Exposition of the Symptoms, essential Nature, and Treatment of Neuropathy, or Nervousness. By JAMES MANBY GULLY, M.D. &c. . 471

ART. XI.—The Transactions of the Provincial Medical and Surgical Association. Vol. V. . . . . . . . 477

Mr. JEAFFRESON's Successful Removal of an Ovarian Tumour . . ib.
Mr. HUNT on the Superior Oblique and Abductor Muscles of both Eyes . ib.
Dr. DICK's Remarks on the Unity of Organic Structure . . . 478
Dr. SELWYN's Cases of Encysted Dropsy of the Thyroid Gland . . ib.
Mr. POYSER's Cases and Dissections, in reference to the Uncertainty of Diagnosis ib.
Mr. HAMERTON's Case of Tetanus, successfully treated by Carbonate of Iron ib.
Dr. SHAPTER's curious Case of Incapacity to Articulate, and of Loss of Memory of Languages . . . . . . ib.
Mr. SALTER's Case of Malignant Tumour within the Abdomen . . 479
Dr. NORRIS's Case of Death from Diaphragmatic Hernia . . ib.
Dr. WALKER's Cases of Rheumatic Metastasis . . . ib.
Mr. WINDSOR on the Effects of Chronic Pleuritis . . . ib.
Reports of the Birmingham Eye Infirmary, by R. MIDDLEMORE, Esq.; of the Birmingham Dispensary, by Dr. T. O. WARD; and of the Worcester Infirmary ib.
Messrs. RUMSEY and CEELY on the present Condition of Medical Relief for the Sick Paupers, with Recommendations for an altered and improved System ib.

ART. XII.—Zeitschrift für die Ophthalmologie. Herausg. von Dr. F. A. v. AMMON. 480

A Journal of Ophthalmology. Edited by Dr. AMMON, of Dresden.

ART. XIII.—The Human Brain; its Configuration, Structure, Development, and Physiology: illustrated by References to the Nervous System in the lower Orders of Animals. By SAMUEL SOLLY, Esq. . . . 485

---

### PART SECOND.—Bibliographical Notices.

ART. I.—A Course of Legal Study, addressed to Students and the Profession generally. By DAVID HOFFMAN, Jur. Utr. Doct. Göttingen . . 489

ART. II.—Aretæus of the Causes and Signs of Acute and Chronic Disease. Translated from the Greek, by T. F. REYNOLDS, M.B. F.L.S. &c. . . 490

ART. III.—Dr. ERNST LUDWIG HEIM's Vermischte Medicinische Schriften. Im Auftrage des Verfassers nach hinterlassenen Papieren gesammelt, und herausgegeben von Dr. A. PAETSCH, Ausübendem Artze zu Berlin . 492
The Miscellaneous Medical Works of Dr. ERNST LUDWIG HEIM. Collected and published, at the Author's desire, from his posthumous Papers, by Dr. A. PAETSCH, Medical Practitioner in Berlin.

ART. IV.—Observations on the Topography, Climate, and prevalent Diseases of the Island of Jersey; the Result of Meteorological Observations and general Practice during thirteen Years. By G. S. HOOPER, M.D. . . 494

ART. V.—The American Medical Library and Intelligencer: a concentrated Record of Medical Science and Literature. Edited by R. DUNGLISON, M.D. . 495

ART. VI.—The Philosophy of the Eye; being a familiar Exposition of its Mechanism, and of the Phenomena of Vision, with a View to the Evidence of Design. By JOHN WALKER, Esq. . . . . 496

ART. VII.—The Medical Student; or, Aids to the Study of Medicine: including a Glossary of the Terms of the Science, and of the Mode of Prescribing, Bibliographical Notices of Medical Works, the Regulations of the different Medical Colleges of the Union, &c. By R. DUNGLISON, M.D. &c. . ib.

ART. VIII.—Plates of the Cerebro-Spinal Nerves, with References; for the use of Medical Students. By PAUL B. GODDARD, M.D. . . . 497

PAGE

Art. IX.—A Manual of General Anatomy, by F. J. Meckel, Professor of Anatomy at Halle, &c. Translated from the German into French, with additional Notes, by A. J. L. Jourdan and G. Breschet. Translated from the French, with Notes, by A. S. Doane, a.m. m.d., and others . . 497

Art. X.—Practical Facts in Chemistry; exemplifying the Rudiments, and showing with what Facility the Principles of the Science may be experimentally demonstrated, at a trifling Expense, by means of simple Apparatus and portable Laboratories, more particularly in reference to those by R. Best Ede . 498

---

### Part Third.—Selections from Foreign Journals.

### ANATOMY AND PHYSIOLOGY.

On the Structure of the Retina in Man, and the Mammalia generally. By Dr. C. M. Gottsche, of Altona . . . . . 499

Microscopical Observations on the visible Motion of the Globules of Lymph in the Lymphatics of Tadpoles. By Professor E. H. Weber, of Leipzig . 500

Case of Loss of Power of Volition over some of the Cerebral Nerves. By Dr. Magnus, of Berlin . . . . . . ib.

### PATHOLOGY, PRACTICAL MEDICINE, AND THERAPEUTICS.

On Pneumonia of the Old. By MM. Hourmahn and Dechambre . . 501

On St. Vitus's Dance. By Dr. Stiebel . . . . 505

Report on the Inoculation of Morphine, &c. proposed by Dr. Lafargue. By M. Martin-Solon . . . . . . . 506

On a peculiar Spasmodic Affection of the Fingers. By Dr. Albers, of Berlin . 507

### SURGERY.

On the Relations and Proportions of the Muscles of the Right and Left Sides in Lateral Curvature of the Spine. By Dr. Gunther, of Hamburg . 509

On Suture of the Intestine. By M. Fleury . . . . 512

On the Nature and Treatment of Itch . . . . . . 513

1. On the Influence of the Human Acarus in the Production of Itch. By M. Albin-Gras . . . . . . . ib.
2. On the Nature and Treatment of Itch. By Dr. Pentzlin, of Wismar . 514
3. Comparison of the Results from the homœopathic and the common Treatment of the Itch. By Dr. Klein, Army Surgeon at Stuttgardt . ib.
4. On Solutions of Caustic in Treatment of Scabies, in all its Forms. By M. Malapert. Report of MM. Alibert, Biett, and Bousquet . 515

Case of an entire Division of the Windpipe, with an almost complete Division of the Gullet, cured. By Dr. Michaelsen, of Meldorf . . . ib.

### MIDWIFERY.

On the Puerperal Fever which prevailed in the Lying-in Hospital at Kiel, from September, 1834, to March, 1835, and during the Winter of 1835-6; and on the Treatment by Ice. By Dr. Michaelis . . . 517

Case of Cæsarean Section, performed with success for the fourth time on the same Individual. By Dr. Michaelis, of Kiel . . . . 521

### MEDICAL JURISPRUDENCE.

Cases of Infanticide, with Remarks . . . . . ib.

### MEDICAL STATISTICS.

Statistical Remarks on the Diseases peculiar to Women. By S. Tanchou . 525

### ANIMAL CHEMISTRY.

A Process for discovering Pus in the Blood. By M. Maudl . . 526

### Part Fourth.—Selections from British Journals.

### ANATOMY AND PHYSIOLOGY.

PAGE

On the first Changes in the Ova of the Mammifera, in consequence of Impregnation; and of the Mode of Origin of the Chorion. By T. Wharton Jones, Esq. 527

On the Temperature of Insects, and its Connexion with the Functions of Respiration and Circulation. By George Newport, Esq. . . . ib.

On the Brain of the Negro, compared with that of the European and the Ourang-Outang. By Frederick Tiedemann, M.D. . . . 529

On the Voluntary and Instinctive Actions of Living Beings. By William B. Carpenter, Esq. . . . . . . 530

On Unity of Function in Organized Beings. By Wm. B. Carpenter, Esq. . 532

### PATHOLOGY, PRACTICAL MEDICINE, AND THERAPEUTICS.

An Account of Hernia Pericardii. By T. Hart, A.B. &c., Dublin . . ib.

On the Employment of Tartar Emetic in large Doses in Inflammation of the Mamma. By J. C. W. Leyer, Esq. . . . . . 533

On the Efficacy of the Cold Affusion in the Treatment of Poisoning with Hydrocyanic Acid. By J. T. Banks, M.D., Physician to the Louth Dispensary . ib.

On Violent Pulsations of the Aorta in the Epigastric Region, and their Treatment. By W. Faussett, A.R. Dublin . . . . . ib.

Observations on the Character and Treatment of the Spotted Fever, at present existing in St. Giles's and the Neighbourhood. By John Wilson, M.D. . 535

Report of St. John's Fever and Lock Hospitals, Limerick. By W. J. Geary, M.D. 536

Cure of Gout by Colchicum applied externally. By T. W. Wansbrough, Esq. . ib.

On the Existence of a "Cerebral Murmur" in Chronic Affections of the Brain. By J. R. Smyth, M.D. . . . . . ib.

### SURGERY.

An Inquiry into the Possibility of Transplanting the Cornea, with a View of relieving Blindness. By S. L. L. Bigger, M.B. &c. . . . 537

Observations on the Advantage of Healing by the first Intention the Wound in the Lateral Operation for Lithotomy. By John Crichton, Esq. Dundee . 539

Club Foot successfully treated by the Division of the Tendo Achillis. By G. Ray, Esq. ib.

Treatment of Gonorrhœa in the Female by solid Nitras Argenti. By J. Bell, Esq. 540

### PHARMACY.

New Preparation of Opium. By Joseph Houlton, Esq. . . . ib.

---

### Part Fifth.—Medical Intelligence.

On the State of Medicine in Norway. By Frederick Holst, M.D., Professor of Medicine at the Royal Frederick's University in Christiania . . 541

Fifth Anniversary Meeting of the Provincial Medical and Surgical Association . 551
Provincial Medical Association . . . . . 560
On the Reproductive Process in Plants . . . . 561
Bavarian Universities . . . . . . 562
Graduations in the University of Edinburgh . . . 563
Dr. Lewins on the Administration of Colchicum . . . . 565
British Association for the Advancement of Science . . . 567
Medical Missions to China . . . . . . 568
Medical Staff of the Queen . . . . . . ib.

Death of Mr. Sherwood . . . . . ib.

Books received for Review . . . 569

